# DMAIC‐ing a Difference: Improving Formative Feedback in Clinical Clerkships

**DOI:** 10.1111/tct.70400

**Published:** 2026-03-15

**Authors:** Ajleeta Nestani, Benjamin Beduhn, Carrie Tamarelli, Allison Ruff

**Affiliations:** ^1^ Department of Obstetrics and Gynecology University of Michigan Ann Arbor Michigan USA; ^2^ Department of Family Medicine University of Michigan Ann Arbor Michigan USA; ^3^ Department of Psychiatry University of Michigan Ann Arbor Michigan USA; ^4^ Department of General Medicine University of Michigan Ann Arbor Michigan USA

**Keywords:** clinical rotations, formative feedback, quality improvement\

## Abstract

**Background:**

Formative feedback is integral to the educational process. We recognised the need to improve our formative feedback due to variable quality, timeliness and depth. Our objective was to improve the quantity and quality of formative feedback given to medical students during clinical rotations using the Define‐Measure‐Analyse‐Improve‐Control (DMAIC) problem‐solving method.

**Approach:**

Based on initial assessment of 50% of students receiving completed formative feedback forms within 72 h after request, we created a task force with representatives from each rotation that met biweekly for 1 year. High‐quality feedback was that achieving a Quality of Assessment for Learning score ≥ 4. We identified stakeholders and conducted focus groups to determine barriers; solutions were implemented asynchronously with task force input and iteration as necessary. Formal data were collected upon task force implementation and periodically for a year. Three key deficiencies emerged: faculty education regarding formative feedback, time for completion and proximity of receiving forms to the encounter. Three methods for improvement were implemented at representative discretion: QR codes to access forms, shortened forms with modified verbiage and increased small‐group faculty education.

**Evaluation:**

Although formally collected baseline data indicated a 73.4% completion rate for formative feedback forms (higher than anticipated), which remained unchanged over the course of a year, the interventions significantly increased the frequency of high‐quality feedback (37.5%–59.4%, *p* = 0.01), with improvement in all rotations.

**Implications:**

Implementation of a task force combined with increased education and potentially simplifying form completion and wording improved formative feedback quality while maintaining quantity.

## 1 Introduction

Formative feedback is a crucial component of the educational process—particularly in medical education, where continuous assessment and constructive feedback shape competent healthcare professionals [1]. Effective formative feedback enhances students' knowledge and skills and fosters critical thinking, self‐reflection and professional growth [2, 3]. It allows learners to identify strengths and areas for improvement, contributing to their development as future physicians. Despite its recognised importance, lack of high‐quality and high‐quantity formative feedback is a pervasive issue in clerkship medical education and beyond [1, 4, 5]. Previous methods for improving feedback include a checklist of items that demonstrate competency, artificial intelligence, online assessments and self‐regulated learning—all with varying success and applicability to clerkship medical education [6, 7, 8].

At our institution, we identified a significant gap in the formative feedback process for medical students during their required clinical rotations. Feedback forms are student‐generated forms sent via e‐mail to faculty after an encounter. Only about 50% of requested formative feedback forms were completed, indicating a concerning deficiency in feedback provision. Moreover, the quality of feedback remained unmonitored, leading to inconsistencies in depth, timeliness and effectiveness. Without adequate formative feedback, students may struggle to recognise areas needing improvement, limiting their ability to refine clinical competencies and decision‐making skills. Since clinical rotations are foundational in medical training, addressing these issues is imperative to ensure students receive the guidance they need to develop into proficient, reflective practitioners [9].

The Quality of Assessment for Learning (QuAL) framework is an established tool for evaluating feedback quality [10]. It provides a structured approach to assessing feedback based on criteria such as specificity, relevance, and actionability. Feedback rated with a QuAL score of ≥ 4 is considered high quality (i.e., meeting the necessary standards to support student learning effectively). By utilising this framework, institutions can ensure that feedback is not only timely but also meets essential characteristics of constructive and meaningful guidance. Incorporating QuAL scoring into formative feedback evaluation enables a more standardised approach to assessing and improving feedback practices in medical education.

The purpose of this project was to systematically define and improve the quantity and quality of formative feedback provided to medical students during clinical rotations. The two specific aims were to (1) increase the quantity of completed formative feedback forms to at least a 75% completion rate and (2) increase the quality of formative feedback to at least 75% high‐quality. Through this initiative, we aimed to foster a culture of meaningful feedback that promotes ongoing learning and professional development. This paper outlines the Define‐Measure‐Analyse‐Improve‐Control (DMAIC) process for improving feedback quality, with a focus on specific measures [11].

## 2 Approach

While the DMAIC method originated in manufacturing, its adoption in medical education remains novel [11, 12]. Traditionally, medical education relies on the Plan‐Do‐Study‐Act (PDSA) framework for continuous quality improvement [13, 14, 15], focusing on incremental, cyclical changes. Although theories of formative assessment have been previously described, they lack guidance for implementation. The persistent gap in formative feedback for clinical medical education requires simultaneous, systematic changes.

### 2.1 Define

We defined the primary problem as a low quantity and quality of formative feedback, with the aim of increasing both feedback form completion rates and delivery of high‐quality feedback to at least 75%. This goal guided our innovative exploration of quality improvement models and the use of DMAIC within medical education.

### 2.2 Measure

This quality improvement project was conducted at a single institution among medical students during their required clinical clerkships starting in 2023. Formative feedback was considered complete if submitted within 72 h of being requested and high‐quality by a QuaL score of ≥ 4.

A multidisciplinary task force with representatives from each rotation met biweekly over 1 year to develop and implement strategies. Root cause analysis was carried out with key stakeholders in structured focus groups, facilitating the identification of barriers and the development of potential solutions for faculty and students.

Interventions were iteratively implemented, guided by the task force and included faculty development workshops and standardised feedback templates. Data collection was performed both at baseline and at the end of each rotation throughout the year to assess impact.

As a quality improvement project, this study did not meet criteria for IRB review. All statistical analyses were performed using R (Vienna, Austria). Linear regression was used to determine if percentages changed over time. The *p* < 0.05 was considered statistically significant.

By repurposing the DMAIC framework for medical education, our approach highlights the potential for cross‐disciplinary innovation in strengthening formative feedback practices during clinical training.

## 3 Evaluation

### 3.1 Analyse

Barriers to frequent, high‐quality formative feedback were systematically evaluated through focus group discussions and stakeholder engagement. As summarised in Table 1, both students and faculty identified three major areas of deficiency: limited education about the purpose and value of formative feedback, the time required to complete feedback forms and misalignment in timing between form distribution and the clinical encounter. These insights informed subsequent intervention strategies directed at improving both usage and effectiveness of formative feedback forms.

**TABLE 1:** Perceived barriers to frequent, high‐quality feedback.

Student concerns	Faculty concerns
Students fear giving faculty ‘more work’ by asking for formative feedback.	Faculty are unsure what to write, particularly if they had limited time with the student.
Students worry that faculty completion of formative feedback form will come at the expense of also completing a summative form.	Many demands on faculty schedules yield limited time to fill out forms.
Written feedback is often not actionable or useful.	Faculty lack understanding of formative vs. summative feedback.
Forms and processes are different on each clerkship, making the process challenging.	Requests for formative feedback forms arrive via email and are often lost or overlooked.
	There is no incentive to complete these forms or to complete them well.

### 3.2 Intervene

In response to the analysed barriers, three targeted interventions were introduced for their impact. The introduction of QR codes facilitated immediate and convenient access to feedback forms, aiming to lower completion barriers. Feedback forms were revised for conciseness and to emphasise formative rather than summative feedback addressing both functional efficiency and alignment with educational goals. Due to technological constraints within certain clerkship platforms, these interventions were selectively implemented in rotations where benefit was projected to be highest. Additionally, educational initiatives were expanded in scope, ranging from individual coaching to department‐wide efforts, to reinforce the significance of formative feedback and clarify expectations for its use. These strategies were monitored for usability and engagement across rotations and revised as needed based on stakeholder feedback.

### 3.3 Control

**FIGURE 1:**
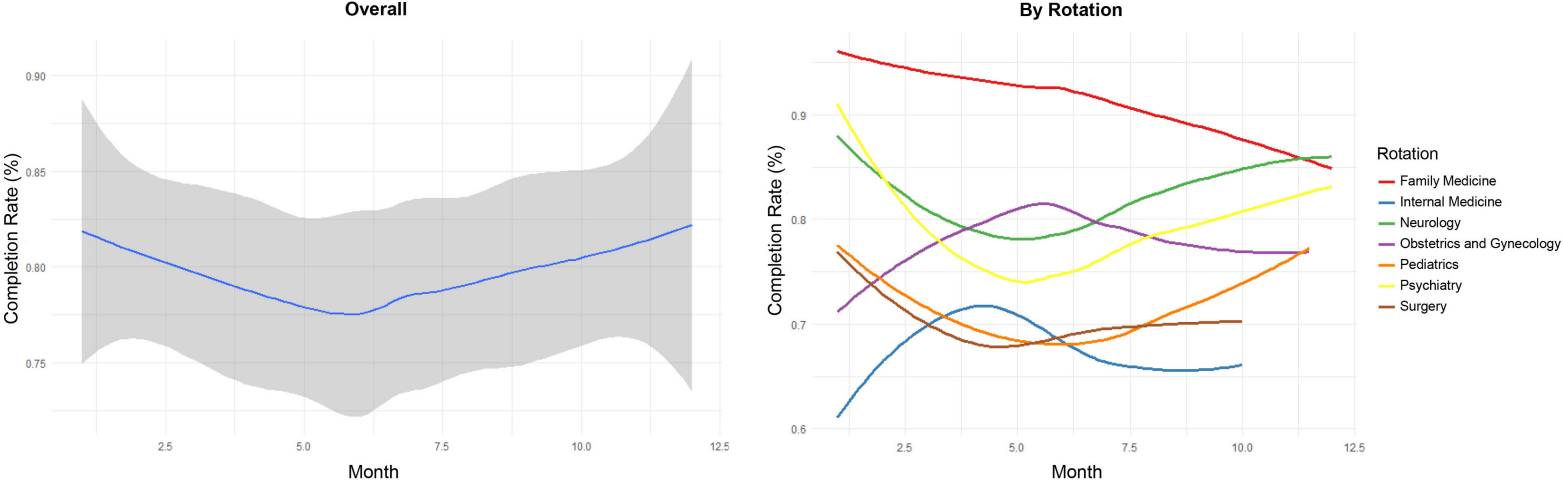
Demonstration of overall form completion rates over 1 year and individual rotation form completion rates.

**FIGURE 2:**
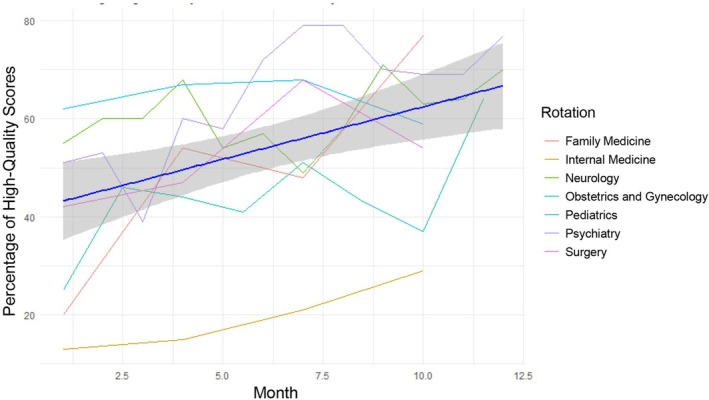
Demonstration of overall percentage of high quality scores over 1 year and individual rotation percentage of high quality scores.

**FIGURE 3:**
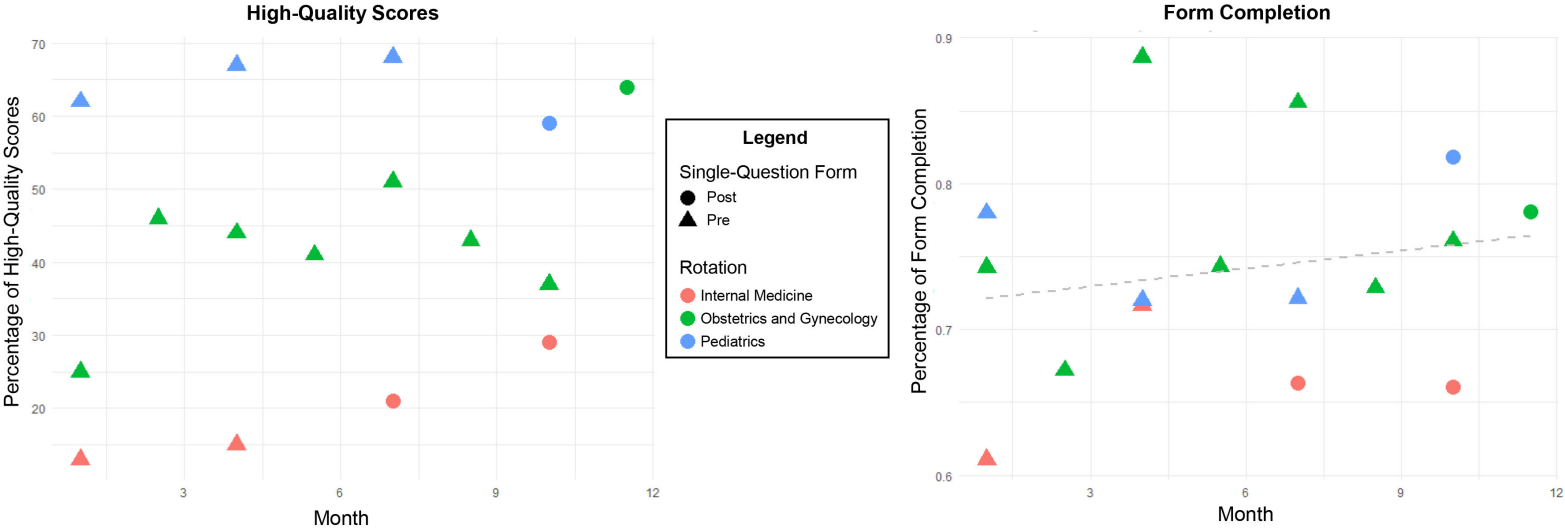
Demonstration of percentage of form completion and high‐quality scores in rotations where single‐question forms were implemented.

Evaluation of sustained impact was conducted by tracking completion rates and feedback quality over the 1‐year period. While overall formative feedback completion rate reached 73.4% (362/493), exceeding initial expectations, no statistically significant change in completion rate was observed during the study period (*p* = 0.37; Figure 1). Notably, the quality of feedback improved substantially, with the percentage of high‐quality feedback rising from 37.5% to 59.4% (*p* < 0.01), and progress was observed consistently across all clinical rotations (Figure 2). All rotations participated in educational initiatives, while the concise feedback form and QR code interventions were adopted in internal medicine, paediatrics and obstetrics and gynaecology at various time points. Although sample sizes for form interventions were limited, preliminary evaluation associated these strategies with improvements in both feedback quality and quantity (Figure 3; detailed form available in the Supporting Information Appendix). Ongoing review of process metrics guided continued adaptation and informed recommendations for broader adoption.

## 4 Implications

Despite multiple interventions, formative feedback completion rates remained steady at 73.4% after 1 year—higher than expected, suggesting strong baseline engagement across rotations. Steady rates may reflect effective mitigation of barriers via QR codes, improved form clarity and targeted education but also indicate the need for additional strategies to boost participation. The consistently high engagement serves as a foundation for ongoing efforts to further enrich formative feedback quality.

The notable rise in high‐quality feedback highlights that interventions supporting clarity and formative intent can meaningfully enhance learning experiences, even without increased completion. Universal education proved evaluators' behaviours are modifiable when feedback goals are clearly communicated.

Applying the DMAIC model facilitated multiple simultaneous improvements, distinguishing this approach from traditional PDSA cycles and demonstrating its feasibility for complex educational projects. Limitations include possible secondary gains from financial incentives for departmental leaders, which, although not directly influencing faculty, may have indirectly promoted best practices. This raises sustainability questions if incentives are discontinued. Additionally, the Hawthorne effect may explain higher initial participation, warranting further study to assess long‐term durability of feedback improvements beyond the incentive period.

## Author Contributions

**Ajleeta Nestani:** conceptualization, data curation, formal analysis, writing‐original draft, writing‐review and editing. **Benjamin Beduhn:** data curation, writing‐review and editing. **Carrie Tamarelli:** data curation, writing‐review and editing. **Allison Ruff:** conceptualization, data curation, methodology, writing‐review and editing.

## Funding

The authors have nothing to report.

## Ethics Statement

This study did not require review by the University of Michigan Institutional Review Board due to its status as a quality improvement project.

## Conflicts of Interest

The authors declare no conflicts of interest.

## Supporting information




**Data S1:** Supporting Information.

## Data Availability

The data that support the findings of this study are available from the corresponding author upon reasonable request.
