# Supplementary figures and images for: DMAIC‐ing a Difference: Improving Formative Feedback in Clinical Clerkships
Source: Clin Teach. 2026 Mar 15;23(2):e70400. doi: 10.1111/tct.70400 (PMC12989470; doi:10.1111/tct.70400)

**Appendix. Sample single-question formative feedback form**


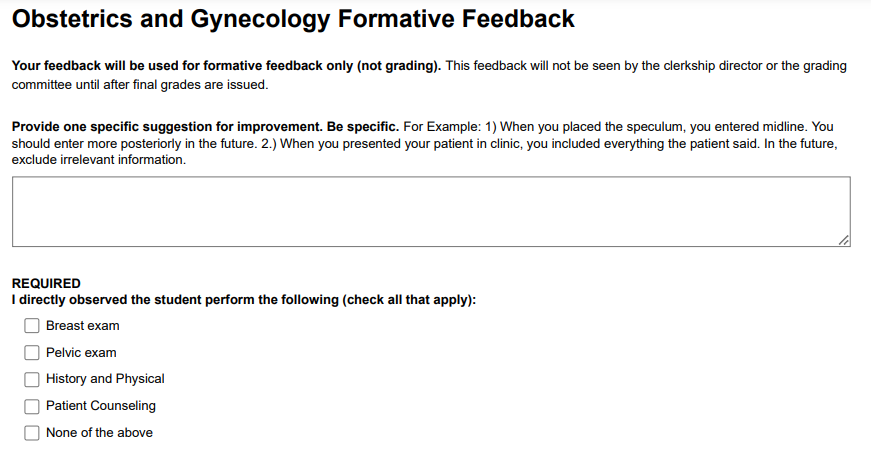

Supplement: Supplementary file 1 — Data S1: Supporting Information. [file TCT-23-e70400-s001.docx]
